# Machine Learning Predicts 30‐Day Readmission and Mortality After Surgical Resection of Head and Neck Cancer

**DOI:** 10.1002/oto2.70100

**Published:** 2025-03-20

**Authors:** Daniel Fu, Aman M. Patel, Lucy Revercomb, Andrey Filimonov, Ghayoour S. Mir

**Affiliations:** ^1^ Department of Otolaryngology–Head and Neck Surgery Rutgers New Jersey Medical School Newark New Jersey USA

**Keywords:** head and neck squamous cell carcinoma, machine learning, mortality, National Cancer Database, readmission, surgical resection

## Abstract

**Objective:**

To develop and validate a machine learning model to identify patients at high risk of 30‐day mortality and hospital readmission using routinely collected health care data.

**Study Design:**

Prognostic predictive modeling and retrospective cohort study. The study was conducted in 2024 using data from 2006 to 2018, with at least a 30‐day follow‐up.

**Setting:**

The 2006 to 2018 National Cancer Database (NCDB).

**Methods:**

The study used deidentified NCDB data on 103,891 head and neck squamous cell carcinoma (HNSCC) patients who underwent surgical resection. Machine learning models were trained on 80% of the data, tested on the remaining 20%, and evaluated using the area under the curve (AUC) and SHapley Additive exPlanations (SHAP) analysis to identify key predictors for 30‐day mortality and readmission.

**Results:**

Among 103,891 patients, 5838 (5.6%) were readmitted, and 829 (0.8%) died within 30 days. The median age was 62, 69% male, and 89% white. Predictors included demographic and clinical data from the NCDB. Five machine learning models were combined and achieved an AUC of 0.80 (95% CI: 0.77‐0.83) for mortality prediction and 0.67 (95% CI: 0.65‐0.68) for readmission prediction. SHAP analysis identified sex and urban‐rural index as key predictors of mortality and readmission, respectively.

**Conclusion:**

Machine learning models can accurately predict mortality and readmission risks, offering insights into the most influential factors. With further validation, these models may enhance clinical decision‐making in postsurgical care for HNSCC patients.

Head and neck squamous cell carcinoma (HNSCC) readmission rates have been reported to range from 7.7% to 26.5%,^1^, ^2^, ^3^ with worse outcomes and a $15,123 increase in costs due to readmission. Previous studies have shown that cancer type, advanced stage, and length of hospitalization are correlated with the risk of hospital readmission^3^, ^4^ but did not provide actionable clinical predictions leveraging these factors to impact clinical decisions. Existing literature has sought to predict HNSCC surgical outcomes such as mortality, readmission, and reoperation rates with varying success.^5^, ^6^, ^7^ To our knowledge, this is the first large‐scale (>100,000 patients) machine learning‐based predictive modeling study focused on predicting 30‐day hospital readmission likelihood following definitive surgical resection for HNSCC using retrospective National Cancer Database (NCDB) clinical data.

A predictive model for mortality and readmission could identify care weak points and target additional resources to high‐risk patients, potentially enhancing decision‐making tools and standard care. This study hypothesizes that retrospective demographic and clinical data can predict 30‐day readmission and mortality. Thus, the objective is to develop and internally validate a machine learning model to assist in decision‐making after HNSCC surgical resection.

## Methods

### Data Set Information

The NCDB, jointly sponsored by the American Cancer Society (ACS) and American College of Surgeons Commission on Cancer (CoC), is a hospital‐based registry that collects data from >1500 CoC‐accredited hospitals within the United States, capturing >70% of patients diagnosed with cancer each year.^8^, ^9^, ^10^ This study was exempt under Rutgers New Jersey Medical School Institutional Review Board review as it involves deidentified patient data. The ACS and CoC are not responsible for the validity of the statistical analysis and conclusions derived herein.

### Study Participant Selection

**Figure 1:**
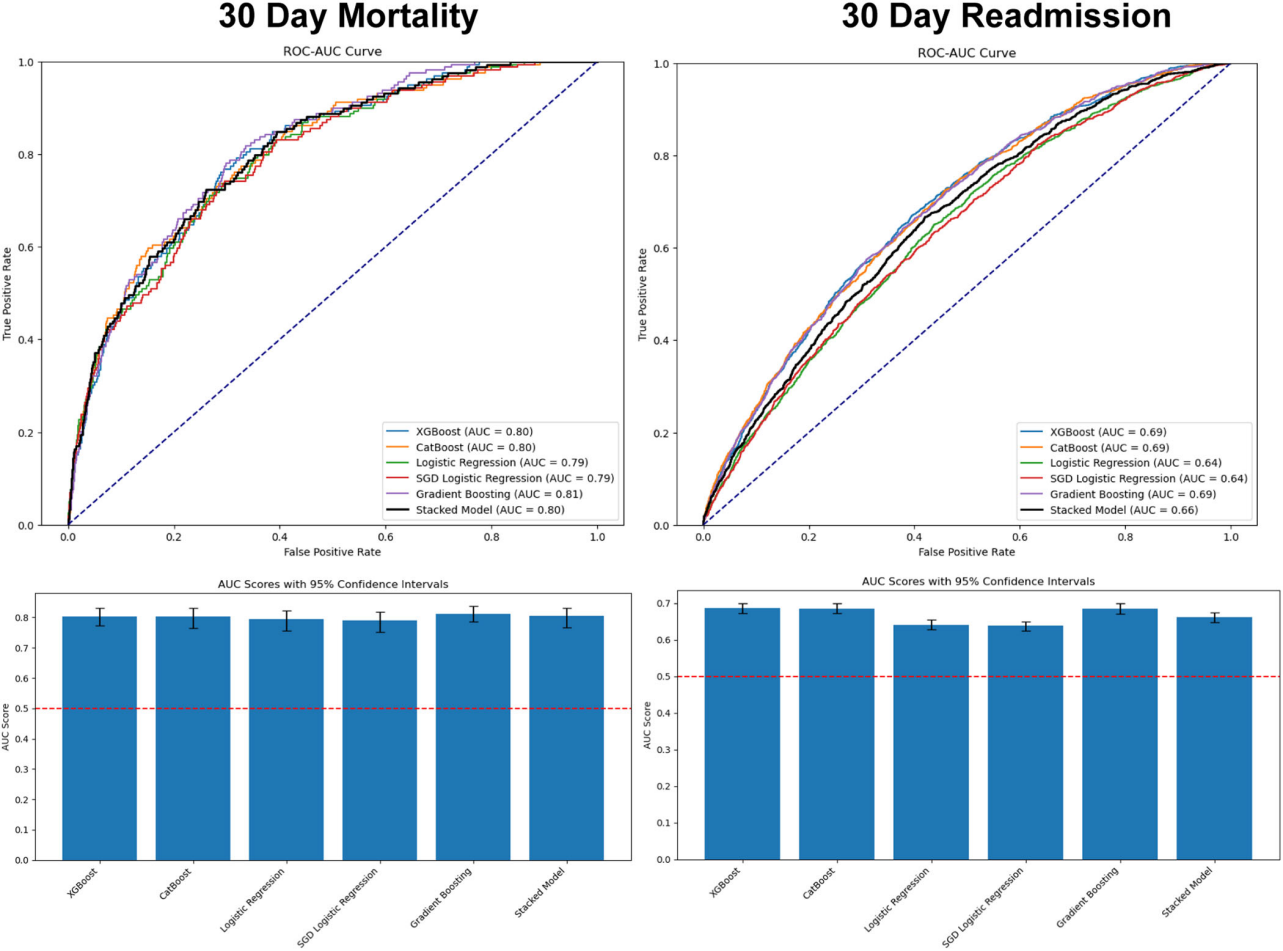
Receiver operating characteristic area under the curve (ROC‐AUC) curves and AUC scores computed on the test set for predicting mortality and readmission rates, showing the performance of five algorithms plus a sixth stacked algorithm.

The 2020 NCDB Participant User File, the most recent PUF available to investigators at the time of the study, was queried on March 1, 2024, for adults with primary HNSCC diagnosed between January 1, 2006, and December 31, 2018, and treated with curative intent with definitive surgical resection (Figure 1). HNSCC was identified using *International Classification of Diseases for Oncology, 3rd Edition* (ICD‐O‐3) histology (“8070,” “8071,” and “8072”), behavior (“3”), and topography (“C00,” “C02,” “C03,” “C04,” “C05,” and “C06” for oral cavity; “C07” and “C08” for major salivary glands; “C11,” “C30.0,” and “C31” for sinonasal tract; “C01,” “C02.4,” “C05.1,” “C05.2,” “C09,” and “C10” for oropharynx; “C12” and “C13” for hypopharynx; and “C32” for larynx) codes. Patients who had diagnosis confirmed without histology; middle ear primary site (“C30.1”), carcinoma in situ; distant metastatic disease; unknown pathologic tumor‐nodal (pTN) classification, 30‐day readmission, 30‐day mortality, or survival time; treatment with palliative intent; salvage surgery; or neoadjuvant radiotherapy or chemotherapy were excluded. A summary of patient selection is visualized by a patient eligibility flowchart in Supplemental Figure S1, available online.

### NCDB Clinical Variables

Patient data included age at diagnosis, sex, race, primary payer status, median household income for the patient's ZIP code of residence according to United States Census data, population density, facility type, location, and volume, travel distance to facility, Charlson‐Deyo comorbidity score (CDCS), history of prior malignancy, primary site, grade, pTN classification, pathologic extranodal extension, lymphovascular invasion, surgical margins, neck dissection, surgical length of stay (LOS), 30‐day readmission and 30‐day mortality following surgical resection, adjuvant therapy, time between surgery and adjuvant radiotherapy (aRT), and other variables shown in Supplemental Table S1, available online. Cases with a CDCS of 0 had no recorded comorbid conditions. Tumors were classified as either low (well or moderately differentiated) or high (poorly differentiated, undifferentiated, or anaplastic) grade. Both macroscopic and microscopic residual tumors were classified as positive surgical margins. Neck dissection was defined as the removal and examination of ≥10 lymph nodes, a threshold validated by previous studies of the NCDB.^11^, ^12^ aRT was defined as the receipt of therapeutic doses (40‐80 Gy) of external beam radiation to the head and neck. The primary outcomes of our study were 30‐day readmission, measured from the date of hospital discharge following surgical resection, and 30‐day mortality, measured from the date of surgical resection.

### Univariate Statistical Analyses

Patients were categorized by 30‐day readmission and 30‐mortality statuses. Univariate analyses comparing patient demographics, clinicopathologic features, and adjuvant therapy were performed using the *χ* ^2^ test and independent samples *t*‐test, as appropriate. All continuous variables were assessed for normality using the Shapiro‐Wilk test. Median, interquartile range, and the Mann‐Whitney *U*‐significance were reported for continuous variables not conforming to a normal distribution. The two‐sided threshold for statistical significance was set at *P* < .05. SPSS version 25 (IBM) was used for univariate statistical analysis.

### Data Set Cleaning and Processing

Data cleaning involved the removal of variables in the NCDB pertaining to nonsurgical treatment or outcomes and the removal of variables with more than 50% missing data. Additionally, missing continuous variables were imputed to the mean, missing ordinal variables were imputed to the median, and missing categorical variables were assigned a new class (missing variable)—otherwise, variables were not recategorized or altered from the NCDB. Further details of how database variables were encoded are available in Supplemental Table S1, available online.

To remove unnecessary, extraneous features and reduce the computational load of model training, Least Absolute Shrinkage and Selection Operator (LASSO) feature reduction was performed (removing all features with coefficients of 0) before model training, which reduced the number of features from 1599 to 102 for mortality and 1599 to 206 for readmission, respectively.

To address class imbalance due to low rates of mortality and readmission, the minority class was upsampled to 17% in the training data set for both outcomes using the Synthetic Minority Over‐sampling TechniquE (SMOTE),^13^ to improve the model's performance for predicting mortality and readmission.

### Model Training

A combination of five machine learning algorithms including linear (logistic regression, stochastic gradient descent logistic regression) and nonlinear algorithms (CatBoost, XGBoost, and Gradient Boosting) were trained to predict the likelihood of 30‐day mortality and 30‐day readmission. See the Supplemental Appendix, available online, for further descriptions of these models. To ensure blinding of algorithmic performance on the test set, the data were randomly split 80:20 train:test n = 83,112, n = 20,779, with clinical variable distributions between the train and test sets shown in Supplemental Table S2, available online. There were no significant differences between the majority of the variables after random splitting.

### Model Testing

Receiver operating characteristic area under the curve (ROC‐AUC) curves were computed for five individual models and the stacked model during cross‐validation/training and testing phases. ROC‐AUC curves visualize the relationship between sensitivity and specificity by plotting sensitivity at various specificity thresholds, generating a smooth curve. An AUC near 0.5 indicates performance no better than random guessing, whereas an AUC of 1.0 indicates perfect accuracy; an AUC of 0.7 is generally considered good. One‐hundred‐fold bootstrapping estimated 95% CIs for AUC values. Model calibration was assessed using a calibration curve. Trained models classified patients into low‐ or high‐risk groups (for death or readmission), with results summarized in confusion matrices.

### Additional Analyses

SHapley Additive exPlanations (SHAP) analysis evaluated the relative importance of features in model predictions, quantifying each feature's impact.^14^ See the Supplemental Appendix, available online, for more on SHAP analysis. Subgroup analyses compared patients above versus below the mean age using the test data set. Subgroups were selected arbitrarily to balance subgroup sizes. Race, sex, and other demographic subanalyses were excluded as LASSO regression eliminated several demographic categories during preprocessing.

Three sensitivity analyses were performed:

1. Training and validation using the full‐featured data set (without removing features with LASSO coefficients of 0) to confirm feature removal did not reduce model performance.
2. Training and validation using principal component analysis (PCA) with 100 components to explore how dimensionality reduction affects performance.
3. Train‐test split by diagnosis date to simulate real‐world testing with pseudo‐retrospective development and pseudo‐prospective validation, ensuring results generalize to future data.

This study was conducted in accordance with Transparent Reporting of a multivariable prediction model for Individual Prognosis Or Diagnosis (TRIPOD) criteria.^15^ The authors are familiar with research conducted on medical databases and the NCDB database variables.

## Results

### Patient Demographics

A summary of participant demographic variables is reported in Table 1. Briefly, patients had a median age of 62, and the majority of patients were male, white, and insured. Tumor grade, lymphovascular invasion, surgical margin positivity, and surgical LOS were significantly higher in the group of patients who were readmitted or died compared to those without readmission or death.

**Table 1:** Patient Demographics, Clinicopathologic Features, and Treatment, by 30‐Day Readmission and 30‐Day Mortality, n (%)^a^

	30‐Day readmission			30‐Day mortality			
	No	Yes	*P* value	No	Yes	*P* value	Total
No.	98,053 (94.4)	5838 (5.6)		103,062 (99.2)	829 (0.8)		103,891
Age at diagnosis, median years (IQR)	62 (54‐71)	62 (54‐70)	**.041**	62 (54‐70)	71 (63‐81)	**<.001**	62 (54‐71)
Sex							
Male	67,831 (69.2)	4085 (70.0)	.201	71,350 (69.2)	566 (68.3)	.553	71,916 (69.2)
Female	30,222 (30.8)	1753 (30.0)		31,712 (30.8)	263 (31.7)		31,975 (30.8)
Race							
White	86,738 (89.3)	5018 (86.8)	**<.001**	91,038 (89.2)	718 (87.7)	**.009**	91,756 (89.2)
Black	6726 (6.9)	520 (9.0)		7168 (7.0)	78 (9.5)		7246 (7.0)
Other	3623 (3.7)	246 (4.3)		3846 (3.8)	23 (2.8)		3869 (3.8)
Primary payer status							
No insurance	3759 (4.0)	277 (4.9)	**<.001**	4016 (4.0)	20 (2.5)	**<.001**	4036 (4.0)
Private insurance, managed care	41,578 (43.8)	2252 (40.1)		43,675 (43.8)	155 (19.4)		43,830 (43.6)
Medicaid	9193 (9.7)	601 (10.7)		9728 (9.8)	66 (8.3)		9794 (9.7)
Medicare	40,427 (42.6)	2484 (44.2)		42,353 (42.4)	558 (69.8)		42,911 (42.7)
Household income							
<$40,227	15,621 (18.4)	1038 (20.7)	**<.001**	16,486 (18.5)	173 (23.8)	**<.001**	16,659 (18.6)
$40,227‐50,353	19,612 (23.1)	1113 (22.2)		20,525 (23.0)	200 (27.5)		20,725 (23.1)
$50,354‐63,332	19,958 (23.5)	1114 (22.2)		20,923 (23.5)	149 (20.5)		21,072 (23.5)
≥$63,333	29,588 (34.9)	1752 (34.9)		31,134 (35.0)	206 (28.3)		31,340 (34.9)
Population density							
Metro	76,355 (82.0)	4677 (83.3)	**.016**	80,411 (82.1)	621 (78.3)	**.006**	81,032 (82.1)
Urban, rural	16,770 (18.0)	940 (16.7)		17,538 (17.9)	172 (21.7)		17,710 (17.9)
Facility type							
Academic	55,891 (58.9)	3562 (62.9)	**<.001**	58,895 (59.0)	558 (67.7)	**<.001**	59,453 (59.1)
Nonacademic	39,030 (41.1)	2102 (37.1)		40,886 (41.0)	266 (32.3)		41,132 (40.9)
Facility location							
New England	4347 (4.6)	267 (4.7)	**<.001**	4584 (4.6)	30 (3.6)	.171	4614 (4.6)
Middle and South Atlantic	33,778 (35.6)	2149 (37.9)		35,645 (35.7)	282 (34.2)		35,927 (35.7)
Central	41,259 (43.5)	2600 (45.9)		43,471 (43.6)	388 (47.1)		43,859 (43.6)
Mountain, Pacific	15,537 (16.4)	648 (11.4)		16,061 (16.1)	124 (15.0)		16,185 (16.1)
Facility volume, median annual cases (IQR)	26.4 (6.2‐52.2)	26.4 (7.9‐53.5)	**.001**	26.4 (6.2‐52.2)	33.1 (12.5‐61.0)	**<.001**	26.4 (6.3‐53.5)
Travel distance to facility, median mi (IQR)	17.0 (6.9‐43.7)	15.3 (6.5‐42.2)	**<.001**	16.9 (6.9‐43.5)	22.7 (8.2‐56.9)	**<.001**	16.9 (6.9‐43.6)
CDCS							
0	73,439 (74.9)	4206 (72.0)	**<.001**	77,111 (74.8)	534 (64.4)	**<.001**	77,645 (74.7)
≥1	24,614 (25.1)	1632 (28.0)		25,951 (25.2)	295 (35.6)		26,246 (25.3)
History of prior malignancy							
No	70,016 (71.4)	4056 (69.5)	**.001**	73,483 (71.3)	589 (71.0)	.868	74,072 (71.3)
Yes	28,019 (28.6)	1782 (30.5)		29,561 (28.7)	240 (29.0)		29,801 (28.7)
Primary site							
Oral cavity	52,176 (53.2)	2838 (48.6)	**<.001**	54,561 (52.9)	453 (54.6)	**<.001**	55,014 (53.0)
Major salivary glands	3009 (3.1)	124 (2.1)		3105 (3.0)	28 (3.4)		3133 (3.0)
Sinonasal tract	2481 (2.5)	154 (2.6)		2610 (2.5)	25 (3.0)		2635 (2.5)
Oropharynx	24,894 (25.4)	1521 (26.1)		26,262 (25.5)	153 (18.5)		26,415 (25.4)
Hypopharynx	2078 (2.1)	199 (3.4)		2248 (2.2)	29 (3.5)		2277 (2.2)
Larynx	13,415 (13.7)	1002 (17.2)		14,276 (13.9)	141 (17.0)		14,417 (13.9)
Grade							
Low	63,795 (73.4)	3743 (70.7)	**<.001**	67,015 (73.3)	523 (69.4)	**.016**	67,538 (73.2)
High	23,117 (26.6)	1548 (29.3)		24,434 (26.7)	231 (30.6)		24,665 (26.8)
pT classification							
1	36,625 (37.4)	1747 (29.9)	**<.001**	38,222 (37.1)	150 (18.1)	**<.001**	38,372 (36.9)
2	27,804 (28.4)	1641 (28.1)		29,255 (28.4)	190 (22.9)		29,445 (28.3)
3	12,088 (12.3)	820 (14.0)		12,728 (12.3)	180 (21.7)		12,908 (12.4)
4	21,536 (22.0)	1630 (27.9)		22,857 (22.2)	309 (37.3)		23,166 (22.3)
pN classification							
0	52,148 (53.2)	2709 (46.4)	**<.001**	54,446 (52.8)	411 (49.6)	**.004**	54,857 (52.8)
1	14,720 (15.0)	955 (16.4)		15,561 (15.1)	114 (13.8)		15,675 (15.1)
2	29,031 (29.6)	1993 (34.1)		30,751 (29.8)	273 (32.9)		31,024 (29.9)
3	2154 (2.2)	181 (3.1)		2304 (2.2)	31 (3.7)		2335 (2.2)
pENE							
No	49,158 (77.6)	2959 (74.1)	**<.001**	51,734 (77.5)	383 (70.7)	**<.001**	52,117 (77.4)
Yes	14,182 (22.4)	1034 (25.9)		15,057 (22.5)	159 (29.3)		15,216 (22.6)
LVI							
No	50,876 (77.0)	3009 (75.2)	**.011**	53,486 (76.9)	399 (68.2)	**<.001**	53,885 (76.9)
Yes	15,233 (23.0)	992 (24.8)		16,039 (23.1)	186 (31.8)		16,225 (23.1)
Surgical margins							
Negative	79,543 (84.3)	4665 (82.7)	**.001**	83,573 (84.3)	635 (78.9)	**<.001**	84,208 (84.2)
Positive	14,780 (15.7)	973 (17.3)		15,583 (15.7)	170 (21.1)		15,753 (15.8)
Neck dissection							
No	28,097 (28.9)	1228 (21.2)	**<.001**	29,135 (28.5)	190 (23.0)	**<.001**	29,325 (28.5)
Yes	69,128 (71.1)	4556 (78.8)		73,049 (71.5)	635 (77.0)		73,684 (71.5)
Surgical LOS, d (IQR)	4 (1‐8)	6 (2‐10)	**<.001**	4 (1‐8)	8 (4‐13)	**<.001**	4 (1‐8)
Adjuvant therapy							
None	44,218 (55.7)	2500 (53.9)	**<.001**	45,908 (55.2)	810 (99.8)	**<.001**	46,718 (55.6)
aRT alone	17,928 (22.6)	1012 (21.8)		18,940 (22.8)	0 (0.0)		18,940 (22.5)
aCRT	17,253 (21.7)	1128 (24.3)		18,379 (22.1)	2 (0.2)		18,381 (21.9)
Time between surgery and aRT, d							
≤42	17,715 (35.3)	915 (29.6)	**<.001**	18,630 (34.9)	‐	‐	18,630 (34.9)
>42	32,527 (64.7)	2175 (70.4)		34,702 (65.1)	‐	‐	34,702 (65.1)

Bold values are statistically significant (*P* < .05).

Abbreviations: aCRT, adjuvant chemoradiotherapy; aRT, adjuvant radiotherapy; CDCS, Charlson‐Deyo comorbidity score; IQR, interquartile range; LOS, length of stay; LVI, lymphovascular invasion; pENE, pathologic extranodal extension; pTN, pathologic tumor‐nodal.

^a^ Total values summing to less than 103,891 patients indicate missing values for that clinical variable.

### Prediction of Mortality and Readmission Rates

The average 30‐day mortality and 30‐day readmission rates were 0.81%:0.77% and 5.66%:5.48% train:test, respectively. After SMOTE, the mortality rates were 17%:0.77%, and readmission rates were 17%:5.48%. The five models plus the stacked model achieved AUCs of 0.79 to 0.81 (combined model: 0.80 [95% CI: 0.77‐0.83]) for predicting mortality and 0.64 to 0.69 (combined model: 0.66 [95% CI: 0.65‐0.68]) for predicting readmission. ROC‐AUC curves and AUC scores on the test data set are shown in Figure 1. The AUC performance was maintained across age subgroups in a subgroup analysis, as shown in Supplemental Figure S3, available online.

**Figure 2:**
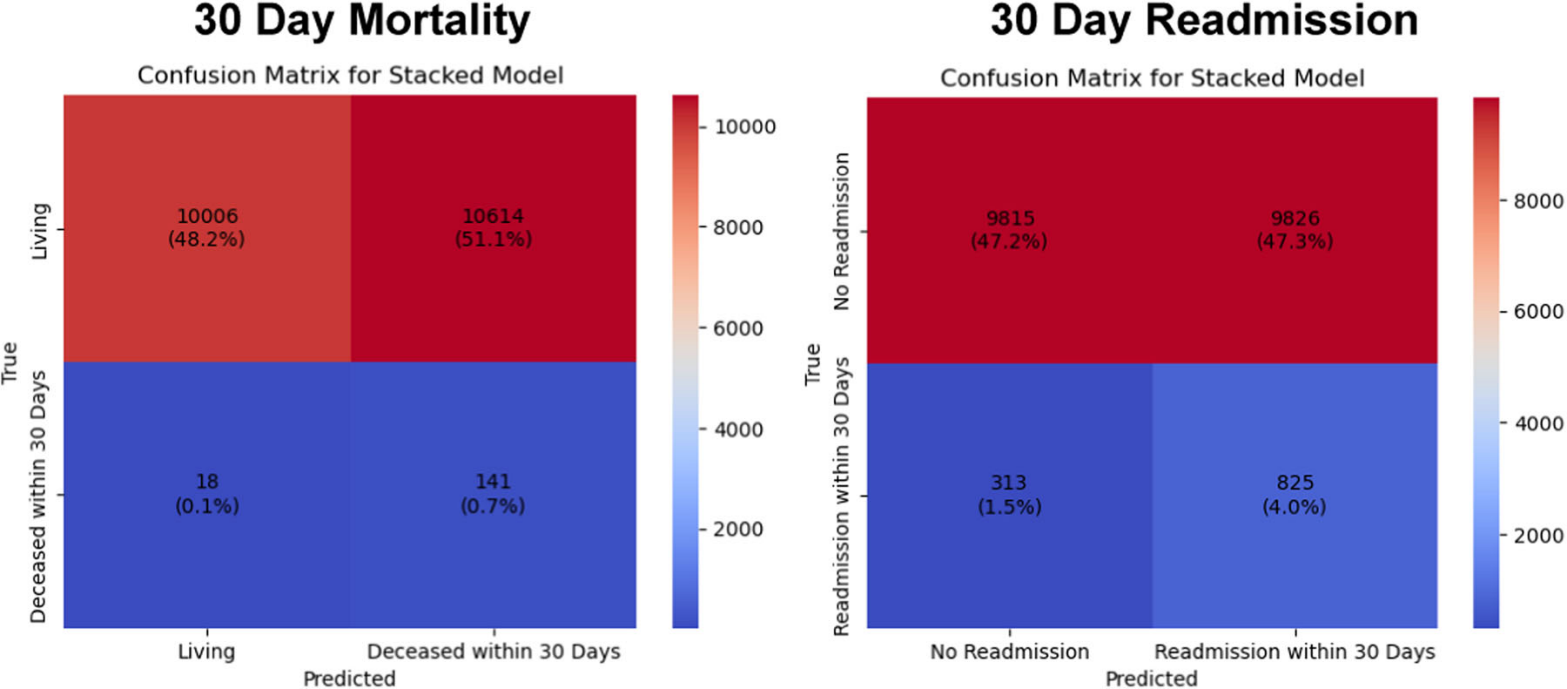
Confusion matrices showing the mortality and readmission versus the model prediction of mortality and readmission.

Using a decision threshold of specificity of 0.5, the combined model had a sensitivity of 0.89 (95% CI: 0.84‐0.93) for predicting mortality and 0.73 (95% CI: 0.70‐0.75) for readmission, with results summarized in confusion matrices in Figure 2. At specificity thresholds of 0.7, sensitivity was 0.72 (95% CI: 0.66‐0.78) for mortality and 0.53 (95% CI: 0.50‐0.56) for readmission.

Using the decision threshold of specificity of 0.5, the combined model was used to separate the test cohort into low‐ and high‐risk groups. The relative risk of an event in the high‐risk versus low‐risk cohort was 7.30 (95% CI: 4.70‐12.21) for mortality (absolute risk 0.01 vs 0.002 for high vs low risk, *P* < .0001) and 2.51 (95% CI: 2.22‐2.80) for readmission (absolute risk 0.08 vs 0.03 for high vs low risk, *P* < .0001).

Models were calibrated on the train data set and were relatively undercalibrated in the test set, with calibration curves provided in Supplemental Figure S2, available online.

### Model Interpretability

**Figure 3:**
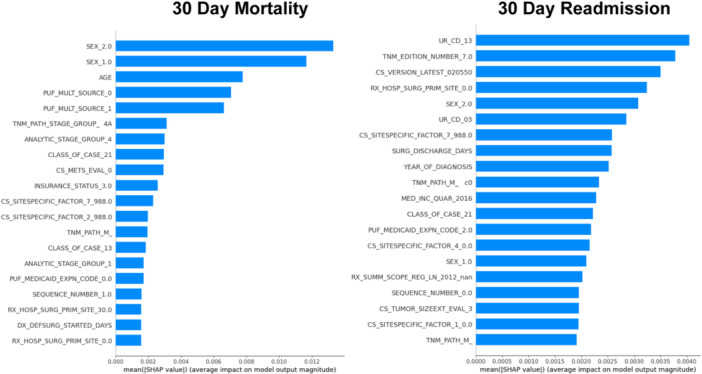
SHapley Additive exPlanations (SHAP) model interpretability analysis of model predictions.

To provide a model interpretability visualization, a SHAP value analysis was performed using the trained stacked model as shown in Figure 3. The features with the highest mean SHAP values for predicting mortality were female sex, male sex, and age versus 2013 urban‐rural index, AJCC Cancer Staging Manual edition number 7, and Collaborative Stage Data Collection System version 2 for predicting readmission.

### Subgroup and Sensitivity Analyses

In the subgroup analysis over the age variable, AUC was similar across all models between the younger and older age subgroups, as shown in Supplemental Figure S3, available online.

**Figure 4:**
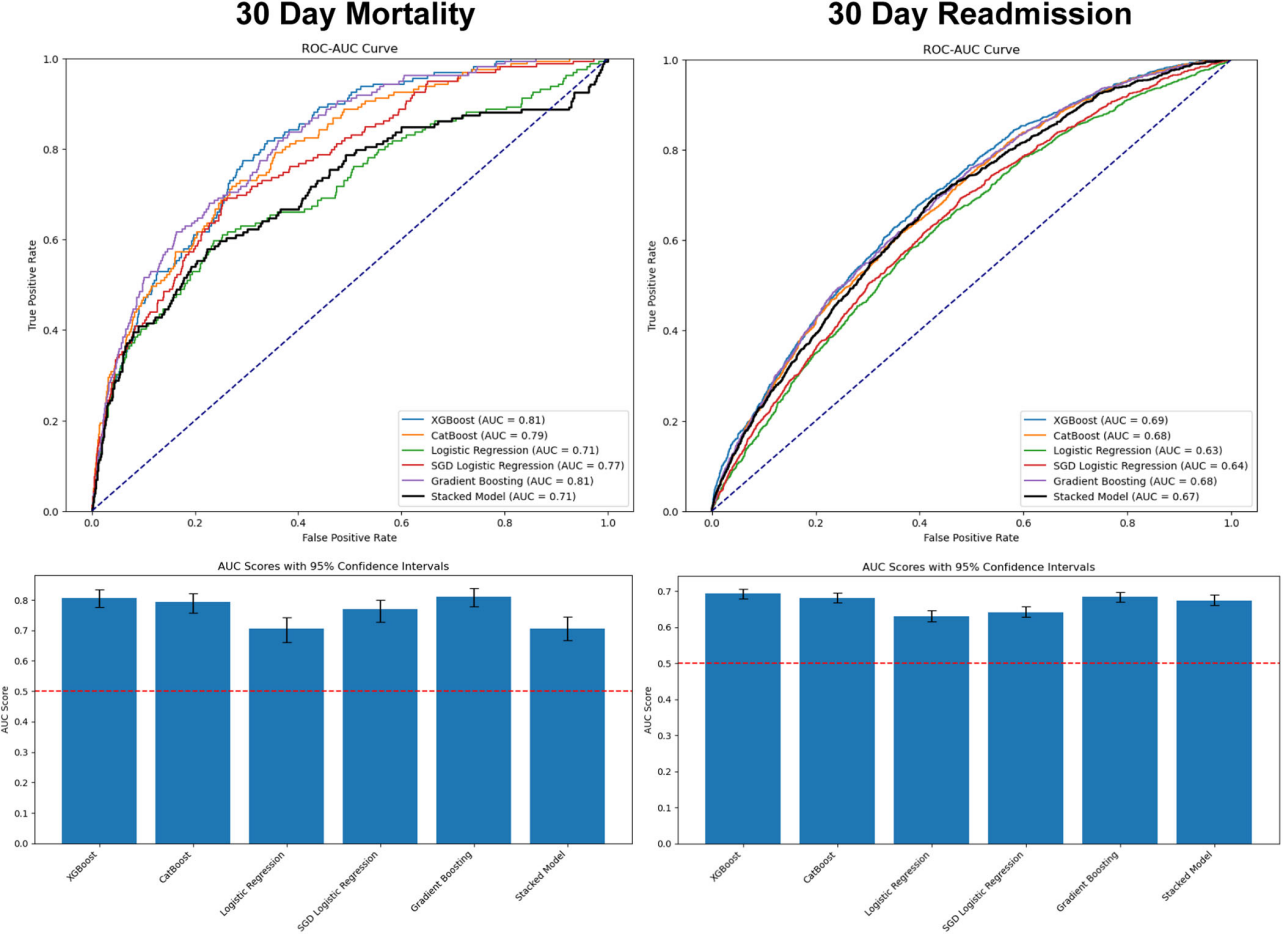
Receiver operating characteristic area under the curve (ROC‐AUC) curves and AUC scores from sensitivity analysis of model performance with inclusion of all features without Least Absolute Shrinkage and Selection Operator feature elimination.

Three sensitivity analyses were performed, including:

1. Model training and validation using the full‐featured training data set, for which the stacked model achieved AUC of 0.71 and 0.67 for mortality and readmission, respectively, compared with an AUC of 0.80 and 0.66 when using the reduced feature set, as shown in Figure 4.
2. Model training and validation using PCA, for which the best model (logistic regression for both) achieved AUC of 0.79 and 0.64 for mortality and readmission, respectively, compared with an AUC of 0.71 and 0.63 when using the original features without PCA, as shown in Supplemental Figure S4, available online.
3. Train:test split by diagnosis date, for which the best model achieved an AUC of 0.75 (XGBoost) and 0.64 (CatBoost) for mortality and readmission, respectively, compared with an AUC of 0.80 and 0.69 when using the random train:test split, as shown in Supplemental Figure S5, available online.

To enhance the reproducibility of this study, the prediction model code, trained models, and code for all additional analyses (Python 3.12.2) have been uploaded to GitHub with the code link provided below.

## Discussion

Machine learning models were highly capable of predicting mortality and readmission rates from retrospective, routinely collected clinical data, with out‐of‐sample test AUCs of 0.80 and 0.66 for the combined model. At a decision threshold with specificity set to 0.5, the combined model had a sensitivity of 0.89 and 0.73 for predicting 30‐day mortality and 30‐day readmission, respectively. In general, selecting a higher specificity threshold results in a lower sensitivity and vice versa. The readmission‐based model is best used to identify patients at high risk for readmission with reasonable sensitivity (0.73), prioritizing avoiding missed cases of potential readmission. Due to the tradeoff of low specificity (0.5), low‐cost interventions, such as patient counseling or electronically flagging patients at high risk, should be considered. The mortality‐based model is best used to target interventions to high‐risk patients, appropriate given its higher sensitivity (0.72) and specificity (0.70). Interventions for high‐risk patients may include more frequent follow‐up visits, referrals to cancer support groups, or additional counseling. Specificity thresholds may be adjusted based on the resource availability of the clinical practice, with high‐resource settings prioritizing sensitivity to benefit more patients and low‐resource settings prioritizing specificity to maximize the benefit of targeted resources.

Models had lower predictive accuracy for readmission than mortality, likely due to label inaccuracy from challenges in documenting readmission across systems, unmeasured factors like transportation or economic barriers, and weaker associations between clinical variables and readmission.

LASSO regression improved computational efficiency, avoided overfitting, and maintained model performance. SHAP analysis highlighted sex and age as key for mortality predictions, whereas the urban‐rural index, AJCC staging, and data collection version were critical for readmission. The staging edition and data collection version likely serve as proxies for time, reflecting temporal changes in outcomes and reporting.

Several prior studies have used machine learning to analyze large national databases for predicting oncologic outcomes. For example, models predicting readmission following free flap reconstruction achieved AUC = 0.78 using XGBoost on a smaller data set (n = 4333) with more detailed patient‐level data (eg, lab values, comorbidities) from the National Surgical Quality Improvement Program (NSQIP) dataset.^6^ Another study achieved AUC = 0.87 for readmission prediction using Random Forest + XGBoost on elderly patients in China (n = 10,358) but required labor‐intensive manual curation.^5^ In a study predicting the detection of metastases in sentinel lymph node biopsy for cutaneous head and neck melanoma, an of AUC = 0.73 was achieved, with validation on an external data set, although the data set size was small (n = 96).^16^ A study investigating the use of machine learning to predict survival following chemoradiation for head and neck cancer achieved a hazard ratio of 0.79 to 0.90 on internal validation,^17^ whereas a similar survival study demonstrated AUC = 0.8^18^; however, neither study assessed readmission rate or was validated on an independent validation set.

Similar predictive models for outcomes such as delayed radiation therapy (accuracy = 64%),^19^ prolonged radiation therapy (AUC = 0.53‐0.66),^20^ postoperative complications (AUC = 0.61)^7^ (another study accuracy = 65%‐75%),^21^ salvage total laryngectomy (AUC = 0.76),^19^ extracapsular extension (AUC = 0.58‐0.68),^22^ and occult nodal metastasis (AUC = 0.66)^23^ further highlight the modest predictive capacity of ML models in this context, with most achieving AUCs around 0.6 to 0.7. Our study differs in focusing specifically on HNSCC, a population with unique clinical challenges, and includes mortality prediction, which provides valuable clinical utility despite modest readmission performance. Although our reliance on NCDB limits detailed patient‐level features, the large sample size and focus on head and neck cancer enhance the generalizability of our findings, and to the author's knowledge, this is the largest study investigating mortality and readmission rates in the United States for HNSCC to date.

This study had several limitations, including lack of data set linkage, potentially leading to exclusion of important clinical variables (eg. smoking and p16 status), unmeasured confounding due to NCDB excluding certain subpopulations, exclusion or imputation of some missing variables, leading to loss of data and a minor potential decrease in model performance, reliance on NCDB coding, the quality of which is maintained by the American College of Surgeons,^24^ use of “site‐specific factors” which are not required to be collected or submitted to the NCDB; thus, these variables may be more prone to inaccuracies or missingness. However, the large majority of site‐specific variables were not important for model predictions as evidenced by low SHAP value magnitude and thus likely had a negligible negative impact on model performance. Models were relatively undercalibrated for mortality likely due to a low number of cases, but reasonably calibrated for readmission due to a higher proportion of cases, as shown in Supplemental Figure S2, available online. To address this limitation, SMOTE was employed to oversample the minority class, leading to a modest improvement in model performance (data not shown). Lastly, the stacked model had reduced performance in the PCA sensitivity analyses, likely due to the loss of predictive information during feature transformation.

Another limitation of this study is the lack of an external test data set. To address this, a time‐based train:test split subanalysis was performed, training on data from 2006 to 2015 and testing on data from 2016 to 2018, simulating an independent data set. This analysis showed AUCs of 0.75 for mortality and 0.64 for readmission, demonstrating robustness to temporal changes. However, external validation remains essential to confirm these results and will be the focus of future studies. Despite these limitations, the size and scope of this study suggest that the model's results are likely generalizable to future NCDB cohorts in the United States, barring substantial shifts in underlying distributions.

## Conclusion

This machine learning model accurately predicts 30‐day readmission and mortality following HNSCC surgical resection, supporting clinical decisions postsurgery using routinely collected data. Identifying high‐risk patients at discharge may guide interventions like patient navigation or increased follow‐ups to reduce adverse outcomes. Future studies should focus on external validation using independent data sets, real‐world testing, developing clinical guidelines, and evaluating model efficacy in practice.

## Author Contributions

**Daniel Fu**, conception, design, analysis, interpretation, manuscript writing; **Aman M. Patel**, conception, design, analysis, interpretation, manuscript writing; **Lucy Revercomb**, design, analysis, interpretation, manuscript writing; **Andrey Filimonov**, design, analysis, interpretation, manuscript writing, final approval; **Ghayoour S. Mir**, design, analysis, interpretation, manuscript writing, final approval.

## Disclosures

### Competing interests

None.

### Funding source

None.

## Supporting information

Supporting information.

Supporting information.

Supporting information.

Supporting information.

Supporting information.

Supporting information.

## Data Availability

Data from the National Cancer Database used in this study are only available through an application process to investigators associated with CoC‐accredited cancer programs, as described at this link: https://www.facs.org/quality-programs/cancer-programs/national-cancer-database/puf/. To use this model, please refer to the attached ReadMe on GitHub, which is also copied in the Supplement.
