# Supplementary figures and images for: Machine Learning Predicts 30‐Day Readmission and Mortality After Surgical Resection of Head and Neck Cancer
Source: OTO Open. 2025 Mar 20;9(1):e70100. doi: 10.1002/oto2.70100 (PMC11924807; doi:10.1002/oto2.70100)

**Figure S1.** Eligibility flowchart with study inclusion and exclusion criteria


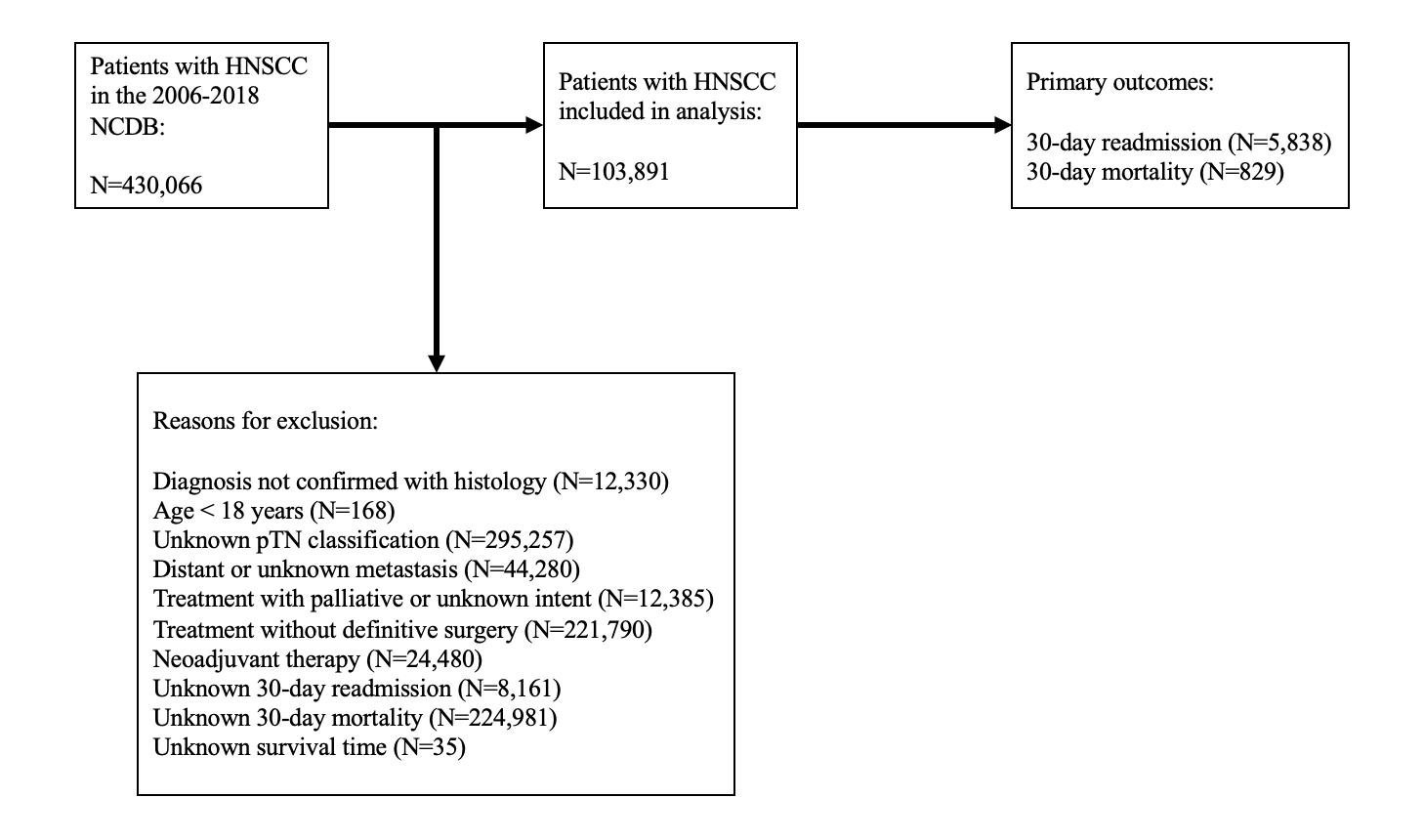

Supplement: Supplementary file 1 — Supporting information. [file OTO2-9-e70100-s003.docx]

**Figure S2.** Model calibration curve

**30 Day Mortality 30 Day Readmission**

**
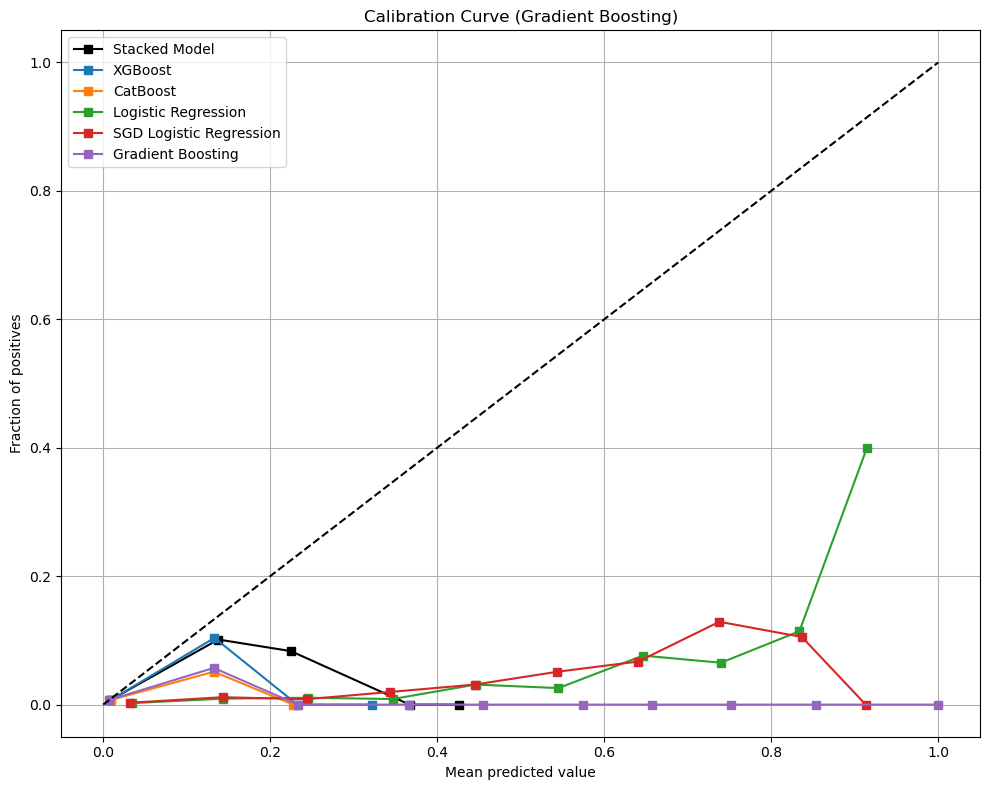

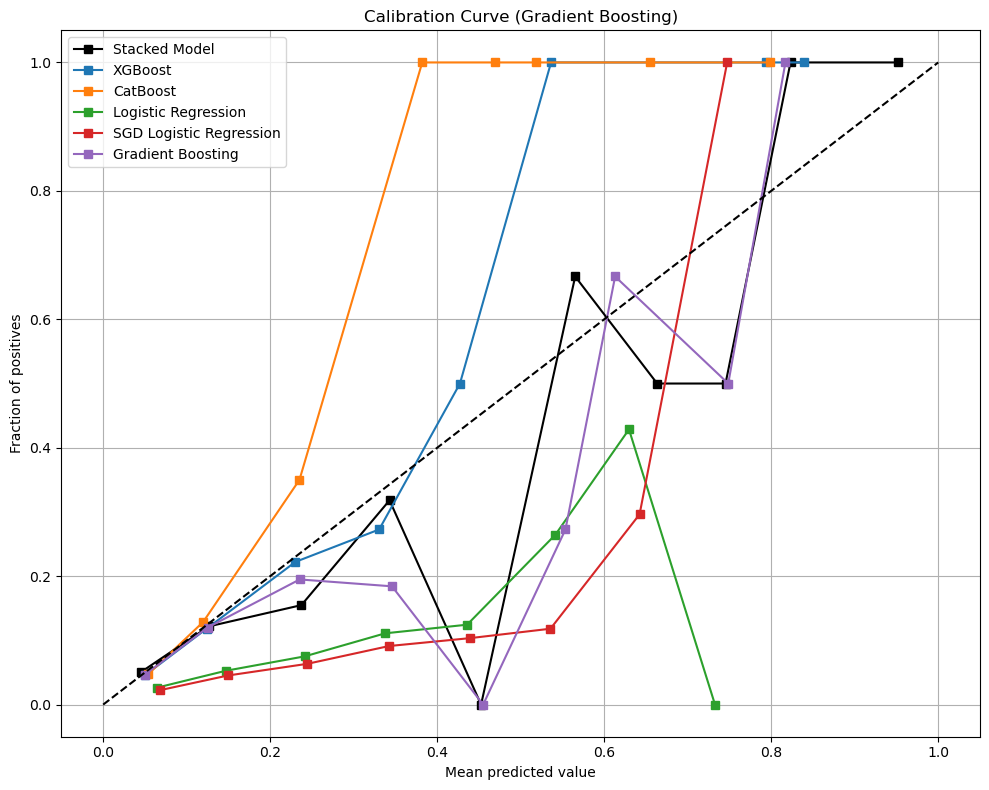
**

Supplement: Supplementary file 2 — Supporting information. [file OTO2-9-e70100-s006.docx]
