# Supplementary material for: Machine Learning Predicts 30‐Day Readmission and Mortality After Surgical Resection of Head and Neck Cancer
Source: OTO Open. 2025 Mar 20;9(1):e70100. doi: 10.1002/oto2.70100 (PMC11924807; doi:10.1002/oto2.70100)
Supplement: Supplementary file 4 — Supporting information. [file OTO2-9-e70100-s004.docx]

**Figure S4.** ROC-AUC curves and AUC scores from sensitivity analysis of model performance using PCA for preprocessing of features prior to training.

**30 Day Mortality 30 Day Readmission**

**
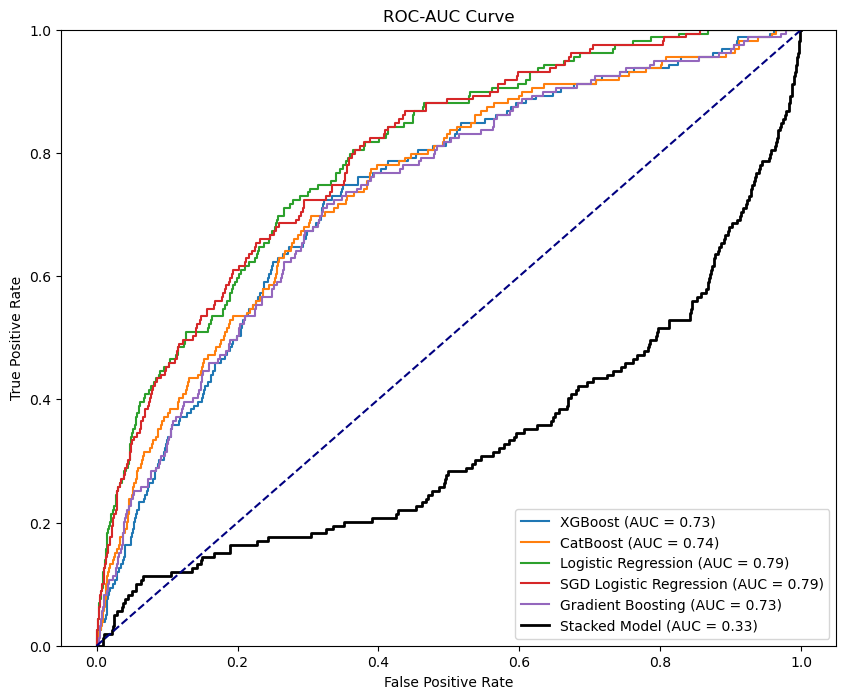

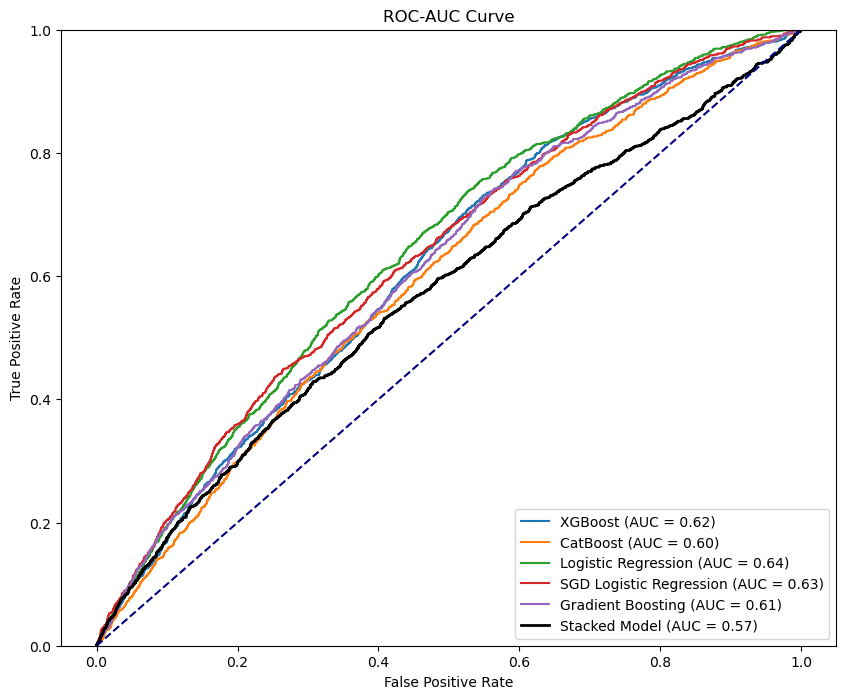
**

**
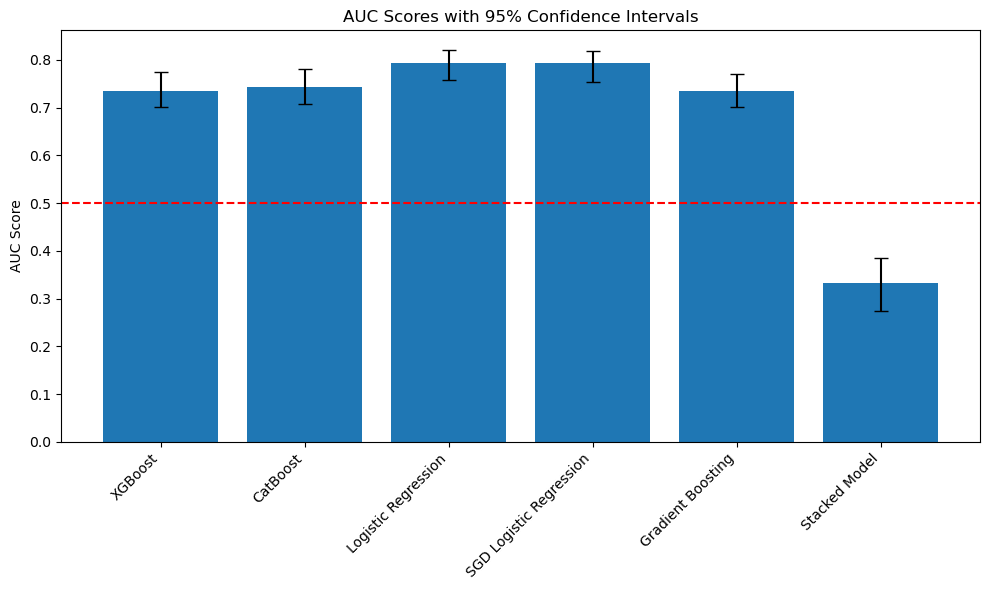

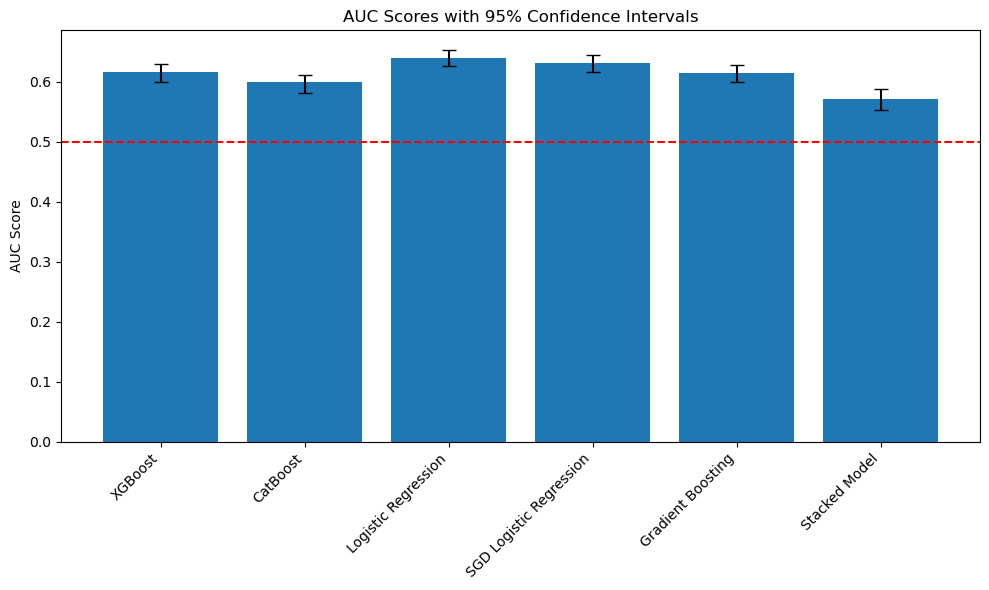
**
