# Supplementary material for: Machine Learning Predicts 30‐Day Readmission and Mortality After Surgical Resection of Head and Neck Cancer
Source: OTO Open. 2025 Mar 20;9(1):e70100. doi: 10.1002/oto2.70100 (PMC11924807; doi:10.1002/oto2.70100)
Supplement: Supplementary file 6 — Supporting information. [file OTO2-9-e70100-s001.docx]

**Supplemental Material for Machine Learning Predicts 30-Day Readmission and Mortality After Surgical Resection of Head and Neck Cancer**

**Table S1.** Table of all data variables, groupings, and preprocessing steps

| **NCDB Variable Name** | **Variable Encoding** | **Reason for Removal** | **Special Considerations** |
| --- | --- | --- | --- |
| AGE | Continuous variables normalized to mean=0 std_dev=1 on the training data. Missing values are imputed to the mean value. | N/A, these variables were included | Values of 999 are coded as “missing” |
| CROWFLY |  |  | Crowfly is distance in mi from nearest hospital |
| YEAR_OF_DIAGNOSIS |  |  |  |
| REGIONAL_NODES_POSITIVE |  |  | Values ≥91 are coded as “missing” |
| DX_STAGING_PROC_DAYS |  |  | Variable represents days between initial dx and surgical staging |
| TUMOR_SIZE_SUMMARY_16 |  |  | Values ≥990 are coded as “missing” |
| TUMOR_SIZE |  |  |  |
| DX_SURG_STARTED_DAYS |  |  | Variable represents days between initial dx and first surgical procedure |
| DX_DEFSURG_STARTED_DAYS |  |  | Variable represents days between initial dx and definitive surgical resection of primary site |
| REGIONAL_NODES_EXAMINED |  |  | Values ≥91 are coded as “missing” |
| NO_HSD_QUAR_00 | Missing ordinal variables are computed to the median value. Otherwise, ordinal variables are retained in their original form. |  | Variable represents high school degree quartiles |
| NO_HSD_QUAR_12 |  |  |  |
| NO_HSD_QUAR_2016 |  |  |  |
| MED_INC_QUAR_00 |  |  | Variable represents income quartiles |
| MED_INC_QUAR_12 |  |  |  |
| MED_INC_QUAR_2016 |  |  |  |
| UR_CD_03 |  |  | Variable represents urban rural index |
| UR_CD_13 |  |  |  |
| CDCC_TOTAL_BEST |  |  | Charlson-Deyo Score, values range from 0 a max value of 3 |
| BEHAVIOR |  |  |  |
| RACE | Categorical variables are one-hot encoded  Variables not listed in this table are encoded as categorical |  |  |
| SEX |  |  |  |
| FACILITY_TYPE_CD |  |  |  |
| FACILITY_LOCATION_CD |  |  |  |
| PUF_MULT_SOURCE |  |  |  |
| PUF_REFERENCE_DATE_FLAG |  |  |  |
| SPANISH_HISPANIC_ORIGIN |  |  |  |
| INSURANCE_STATUS |  |  |  |
| PUF_MEDICAID_EXPN_CODE |  |  |  |
| SEQUENCE_NUMBER |  |  | Variable represents sequence of malignant/non-malignant tumors in lifetime |
| CLASS_OF_CASE |  |  | Variable represents where diagnosis and treatment occurred |
| PRIMARY_SITE |  |  |  |
| LATERALITY |  |  |  |
| GRADE |  |  | The variable contains values from 1-4 (Grade I to Grade IV), values of 9 are coded as “missing” |
| GRADE_CLIN |  |  |  |
| GRADE_PATH |  |  |  |
| GRADE_PATH_POST |  |  |  |
| DIAGNOSTIC_CONFIRMATION |  |  |  |
| RX_SUMM_DXSTG_PROC |  |  |  |
| RX_HOSP_DXSTG_PROC |  |  |  |
| TNM_CLIN_T |  |  |  |
| TNM_CLIN_N |  |  |  |
| TNM_CLIN_M |  |  |  |
| TNM_CLIN_STAGE_GROUP |  |  |  |
| TNM_PATH_T |  |  |  |
| TNM_PATH_N |  |  |  |
| TNM_PATH_M |  |  |  |
| TNM_PATH_STAGE_GROUP |  |  |  |
| TNM_EDITION_NUMBER |  |  |  |
| ANALYTIC_STAGE_GROUP |  |  |  |
| ‘METS_AT_DX’ variants |  |  | Any variables that contain the ‘METS_AT_DX’ phrase are encoded as categorical |
| ‘AJCC_TNM’ variants |  |  | Any variables that contain the ‘AJCC_TNM’ phrase are encoded as categorical |
| ‘CS_SITESPECIFIC_FACTOR’ variants |  |  | Any variables that contain the ‘CS_SITESPECIFIC_FACTOR’ phrase are encoded as categorical |
| CS_VERSION_LATEST |  |  |  |
| CS_EXTENSION |  |  |  |
| CS_TUMOR_SIZEEXT_EVAL |  |  |  |
| LYMPH_VASCULAR_INVASION |  |  |  |
| ‘CS_METS’ variants |  |  | Any variables that contain the ‘CS_METS’ phrase are encoded as categorical |
| RX_SUMM_SURG_PRIM_SITE |  |  |  |
| RX_HOSP_SURG_PRIM_SITE |  |  |  |
| RX_HOSP_SURG_APPR_2010 |  |  |  |
| RX_SUMM_SURGICAL_MARGINS |  |  |  |
| RX_SUMM_SCOPE_REG_LN_SUR |  |  |  |
| RX_SUMM_SCOPE_REG_LN_2012 |  |  |  |
| RX_SUMM_SURG_OTH_REGDIS |  |  |  |
| READM_HOSP_30_DAYS | N/A, excluded | Outcome variable | Outcome #1 |
| PUF_30_DAY_MORT_CD |  |  | Outcome #2 |
| PUF_90_DAY_MORT_CD |  | Prevent data leakage | Variable measured after time of surgery |
| DX_LASTCONTACT_DEATH_MONTHS |  |  |  |
| PUF_VITAL_STATUS |  |  |  |
| SURG_DISCHARGE_DAYS |  |  |  |
| REASON_FOR_NO_SURGERY |  |  |  |
| RX_SUMM_TREATMENT_STATUS |  |  | Treatment summary variable |
| DX_RX_STARTED_DAYS |  |  |  |
| DX_RAD_STARTED_DAYS |  |  | Adjuvant radiation-related variables are excluded as they pertain to information measured after time of surgery |
| RAD_LOCATION_OF_RX |  |  |  |
| PHASE_I_RT_VOLUME |  |  |  |
| PHASE_I_RT_TO_LN |  |  |  |
| PHASE_I_RT_MODALITY |  |  |  |
| PHASE_I_BEAM_TECH |  |  |  |
| PHASE_I_DOSE_FRACT |  |  |  |
| PHASE_I_NUM_FRACT |  |  |  |
| PHASE_I_TOTAL_DOSE |  |  |  |
| PHASE_II_RT_VOLUME |  |  |  |
| PHASE_II_RT_TO_LN |  |  |  |
| PHASE_II_RT_MODALITY |  |  |  |
| PHASE_II_BEAM_TECH |  |  |  |
| PHASE_II_DOSE_FRACT |  |  |  |
| PHASE_II_NUM_FRACT |  |  |  |
| PHASE_II_TOTAL_DOSE |  |  |  |
| PHASE_III_RT_VOLUME |  |  |  |
| PHASE_III_RT_TO_LN |  |  |  |
| PHASE_III_RT_MODALITY |  |  |  |
| PHASE_III_BEAM_TECH |  |  |  |
| PHASE_III_DOSE_FRACT |  |  |  |
| PHASE_III_NUM_FRACT |  |  |  |
| PHASE_III_TOTAL_DOSE |  |  |  |
| NUMBER_PHASES_RAD_RX |  |  |  |
| RAD_RX_DISC_EARLY |  |  |  |
| TOTAL_DOSE |  |  |  |
| RX_SUMM_SURGRAD_SEQ |  |  |  |
| RAD_ELAPSED_RX_DAYS |  |  |  |
| REASON_FOR_NO_RADIATION |  |  |  |
| DX_SYSTEMIC_STARTED_DAYS |  |  | Adjuvant systemic therapy-related variables are excluded as they pertain to information measured after the time of surgery |
| RX_SUMM_CHEMO |  |  |  |
| RX_HOSP_CHEMO |  |  |  |
| DX_CHEMO_STARTED_DAYS |  |  |  |
| RX_SUMM_HORMONE |  |  |  |
| RX_HOSP_HORMONE |  |  |  |
| DX_HORMONE_STARTED_DAYS |  |  |  |
| RX_SUMM_IMMUNOTHERAPY |  |  |  |
| RX_HOSP_IMMUNOTHERAPY |  |  |  |
| DX_IMMUNO_STARTED_DAYS |  |  |  |
| RX_SUMM_TRNSPLNT_ENDO |  |  |  |
| RX_SUMM_SYSTEMIC_SUR_SEQ |  |  |  |
| RX_SUMM_OTHER |  |  | Other treatment-related variables are excluded as they pertain to information measured after the time of surgery |
| RX_HOSP_OTHER |  |  |  |
| DX_OTHER_STARTED_DAYS |  |  |  |
| PALLIATIVE_CARE |  |  |  |
| PALLIATIVE_CARE_HOSP |  |  |  |
| REG_LN_DISS_STARTED_DAY |  | Excluded due to missing values |  |
| SURG_DISCHARGE_DAYS |  | Excluded due to missing values |  |

**Table S2.** Patient demographics, clinicopathologic features, and treatment included in training and testing datasets, n (%).

|  | **Training** | **Testing** | ***P* value** |
| --- | --- | --- | --- |
| No. | 83,112 | 20,779 |  |
| Age at diagnosis, median years (IQR) | 62 (54-71) | 62 (54-70) | 0.786 |
| Sex |  |  |  |
| Male | 57,455 (69.1) | 14,461 (69.6) | 0.194 |
| Female | 25,657 (30.9) | 6,318 (30.4) |  |
| Race |  |  |  |
| White | 73,431 (89.2) | 18,325 (89.1) | 0.962 |
| Black | 5,794 (7.0) | 1,452 (7.1) |  |
| Other | 3,090 (3.8) | 779 (3.8) |  |
| Primary payer status |  |  |  |
| No insurance | 3,218 (4.0) | 818 (4.1) | 0.064 |
| Private insurance, managed care | 35,020 (43.5) | 8,810 (43.8) |  |
| Medicaid | 7,757 (9.6) | 2,037 (10.1) |  |
| Medicare | 34,470 (42.8) | 8,441 (42.0) |  |
| Household income |  |  |  |
| $<$ $40,227 | 13,311 (18.5) | 3,348 (18.7) | 0.885 |
| $40,227-50,353 | 16,611 (23.1) | 4,114 (23.0) |  |
| $50,354-63,332 | 16,858 (23.4) | 4,214 (23.6) |  |
| $\geq$ $63,333 | 25,127 (34.9) | 6,213 (34.7) |  |
| Population density |  |  |  |
| Metro | 64,876 (82.1) | 16,156 (81.9) | 0.464 |
| Urban, rural | 14,136 (17.9) | 3,574 (18.1) |  |
| Facility type |  |  |  |
| Academic | 47,584 (59.2) | 11,869 (58.9) | 0.541 |
| Non-academic | 32,856 (40.8) | 8,276 (41.1) |  |
| Facility location |  |  |  |
| New England | 3,709 (4.6) | 905 (4.5) | 0.567 |
| Middle and South Atlantic | 28,796 (35.8) | 7,131 (35.4) |  |
| Central | 35,027 (43.5) | 8,832 (43.8) |  |
| Mountain, Pacific | 12,908 (16.0) | 3,277 (16.3) |  |
| Travel distance to facility, median mi (IQR) | 17.0 (6.9-43.7) | 16.7 (6.8-43.4) | 0.250 |
| CDCS |  |  |  |
| 0 | 62,050 (74.7) | 15,595 (75.1) | 0.243 |
| $\geq$ 1 | 21,062 (25.3) | 5,184 (24.9) |  |
| History of prior malignancy |  |  |  |
| No | 59,201 (71.2) | 14,871 (71.6) | 0.334 |
| Yes | 23,897 (28.8) | 5,904 (28.4) |  |
| Primary site |  |  |  |
| Oral cavity | 43,984 (52.9) | 11,030 (53.1) | 0.932 |
| Major salivary glands | 2,518 (3.0) | 615 (3.0) |  |
| Sinonasal tract | 2,092 (2.5) | 543 (2.6) |  |
| Oropharynx | 21,141 (25.4) | 5,274 (25.4) |  |
| Hypopharynx | 1,832 (2.2) | 445 (2.1) |  |
| Larynx | 11,545 (13.9) | 2,872 (13.8) |  |
| Grade |  |  |  |
| Low | 53,990 (73.2) | 13,548 (73.4) | 0.665 |
| High | 19,749 (26.8) | 4,916 (26.6) |  |
| pT classification |  |  |  |
| 1 | 30,663 (36.9) | 7,709 (37.1) | 0.947 |
| 2 | 23,575 (28.4) | 5,870 (28.2) |  |
| 3 | 10,323 (12.4) | 2,585 (12.4) |  |
| 4 | 18,551 (22.3) | 4,615 (22.2) |  |
| pN classification |  |  |  |
| 0 | 43,971 (52.9) | 10,886 (52.4) | 0.255 |
| 1 | 12,462 (15.0) | 3,213 (15.5) |  |
| 2 | 24,795 (29.8) | 6,229 (30.0) |  |
| 3 | 1,884 (2.3) | 451 (2.2) |  |
| pENE |  |  |  |
| No | 41,789 (77.5) | 10,328 (77.2) | 0.468 |
| Yes | 12,160 (22.5) | 3,056 (22.8) |  |
| LVI |  |  |  |
| No | 43,033 (76.7) | 10,852 (77.5) | **0.032** |
| Yes | 13,082 (23.3) | 3,143 (22.5) |  |
| Surgical margins |  |  |  |
| Negative | 67,337 (84.2) | 16,871 (84.3) | 0.673 |
| Positive | 12,620 (15.8) | 3,133 (15.7) |  |
| Neck dissection |  |  |  |
| No | 23,446 (28.5) | 5,879 (28.5) | 0.871 |
| Yes | 58,945 (71.5) | 14,739 (71.5) |  |
| Surgical LOS, days (IQR) | 4 (1-8) | 4 (1-8) | 0.382 |

Abbreviations: CDCS, Charlson-Deyo comorbidity score; LOS, length of stay; LVI, lymphovascular invasion; pENE, pathologic extranodal extension; pTN, pathologic tumor-nodal; IQR, interquartile range.

**Appendix**

**Supplemental Methods**

Logistic regression and SGD logistic regression use a linear combination of input parameters to draw a straight line that separates different outcomes. These models excel at capturing the combined effect of all the input variables, assuming that the variables are independent. In contrast, CatBoost, XGBoost, and Gradient Boosting are decision tree models, which use multiple, flexible curves or steps to generate predictions. These non-linear models can provide excellent predictions for datasets where variables are dependent and can interact with one another. These particular models were chosen to provide a balanced collection of models while avoiding computationally intensive models such as neural networks, allowing these models to be bootstrapped, nested, and cross-validated without requiring computers with prohibitively high computing power. An aggregate of these models (stacked model) was used to combine the strengths of the individual models to create a single robust predictive model.

Model training involved bootstrapped nested 5-fold cross-validation and the best models were stacked into a single aggregate prediction. As such, models were trained using multiple variations of the data (bootstrapping) to improve accuracy, tested several times (5-fold cross-validation) to find the best model settings (nesting), and then combined to make a single, stronger prediction (stacking).

Briefly, SHAP values are essential for interpreting stacked models that include both linear and nonlinear components because they consistently measure each feature's contribution across different models. Unlike nomograms, ICE, and Partial Dependence Plots, SHAP provides a unified approach that accurately captures feature importance regardless of the underlying model's complexity, offering a more reliable interpretation in mixed-model scenarios.

**ReadMe**

Primary Author

Daniel Fu, MS

Rutgers New Jersey Medical School, Newark, New Jersey, USA

Email: danfu42@gmail.com

Overview

This repository contains Jupyter notebooks for predicting 30-day readmission and 30-day mortality following surgical resection of head and neck squamous cell carcinoma. The analysis is implemented in Python 3.12.2 using Jupyter Notebooks.

Data

The data used in this project is obtained from the National Cancer Database (NCDB). Due to privacy restrictions, the dataset is available by request only. For access to the data, please visit the NCDB Patient User File at https://www.facs.org/quality-programs/cancer-programs/national-cancer-database/puf/.

Installation

Prerequisites

- Python 3.12.2

- Conda environment manager

Setting Up the Environment

1. Clone this repository to your local machine.

2. Navigate to the cloned directory.

3. To create and activate the Conda environment with all the required dependencies, run the following commands:

conda env create -f environment.yaml

conda activate myenv

Usage

Starting Jupyter Notebook

Once the environment is set up, you can start the Jupyter Notebook server using:

jupyter notebook

This command will start the server and open the notebook interface in your default web browser.

Running the Notebooks

The main notebooks for analysis are:

- ent_outcomes_readmission.ipynb for readmission prediction.

- ent_outcomes_mortality.ipynb for mortality prediction.

To run a notebook:

1. Open the notebook in the Jupyter Notebook interface.

2. Navigate to Kernel > Restart & Run All to execute all cells in the notebook.

Note: Running the main notebooks may take up to 12 hours depending on your hardware configuration.

Hardware Specifications

Tested on MacOS Ventura with the following specs:

- Processor: 1.4 GHz Quad-Core Intel Core i5

- Memory: 8 GB 2133 MHz LPDDR3

Additional Information

This repository also includes several utility notebooks and Python pickle files containing intermediate data states to facilitate different stages of analysis. For utility functions used across the project, please refer to ent_utils. ipynb. If you have any questions, please contact Daniel Fu.

License

MIT License

Copyright (c) 2024 Daniel Fu

Permission is hereby granted, free of charge, to any person obtaining a copy of this software and associated documentation files (the "Software"), to deal in the Software without restriction, including without limitation the rights to use, copy, modify, merge, publish, distribute, sublicense, and/or sell copies of the Software, and to permit persons to whom the Software is furnished to do so, subject to the following conditions:

The above copyright notice and this permission notice shall be included in all copies or substantial portions of the Software.

THE SOFTWARE IS PROVIDED "AS IS", WITHOUT WARRANTY OF ANY KIND, EXPRESS OR IMPLIED, INCLUDING BUT NOT LIMITED TO THE WARRANTIES OF MERCHANTABILITY, FITNESS FOR A PARTICULAR PURPOSE AND NONINFRINGEMENT. IN NO EVENT SHALL THE AUTHORS OR COPYRIGHT HOLDERS BE LIABLE FOR ANY CLAIM, DAMAGES OR OTHER LIABILITY, WHETHER IN AN ACTION OF CONTRACT, TORT OR OTHERWISE, ARISING FROM, OUT OF OR IN CONNECTION WITH THE SOFTWARE OR THE USE OR OTHER DEALINGS IN THE SOFTWARE.

**TRIPOD Criteria**

TRIPOD Checklist: <https://www.equator-network.org/reporting-guidelines/tripod-statement/>

TRIPOD Elaboration: <https://www.acpjournals.org/doi/10.7326/M14-0698>

Criteria 17 was not relevant, all other criteria were followed

| **Section/Topic** | **Item** |  | **Checklist Item** | **Page** |
| --- | --- | --- | --- | --- |
| **Title and abstract** | | | | |
| Title | 1 | D;V | Identify the study as developing and/or validating a multivariable prediction model, the target population, and the outcome to be predicted. |  |
| Abstract | 2 | D;V | Provide a summary of objectives, study design, setting, participants, sample size, predictors, outcome, statistical analysis, results, and conclusions. |  |
| **Introduction** | | | | |
| Background and objectives | 3a | D;V | Explain the medical context (including whether diagnostic or prognostic) and rationale for developing or validating the multivariable prediction model, including references to existing models. |  |
|  | 3b | D;V | Specify the objectives, including whether the study describes the development or validation of the model or both. |  |
| **Methods** | | | | |
| Source of data | 4a | D;V | Describe the study design or source of data (e.g., randomized trial, cohort, or registry data), separately for the development and validation data sets, if applicable. |  |
|  | 4b | D;V | Specify the key study dates, including start of accrual; end of accrual; and, if applicable, end of follow-up. |  |
| Participants | 5a | D;V | Specify key elements of the study setting (e.g., primary care, secondary care, general population) including number and location of centres. |  |
|  | 5b | D;V | Describe eligibility criteria for participants. |  |
|  | 5c | D;V | Give details of treatments received, if relevant. |  |
| Outcome | 6a | D;V | Clearly define the outcome that is predicted by the prediction model, including how and when assessed. |  |
|  | 6b | D;V | Report any actions to blind assessment of the outcome to be predicted. |  |
| Predictors | 7a | D;V | Clearly define all predictors used in developing or validating the multivariable prediction model, including how and when they were measured. |  |
|  | 7b | D;V | Report any actions to blind assessment of predictors for the outcome and other predictors. |  |
| Sample size | 8 | D;V | Explain how the study size was arrived at. |  |
| Missing data | 9 | D;V | Describe how missing data were handled (e.g., complete-case analysis, single imputation, multiple imputation) with details of any imputation method. |  |
| Statistical analysis methods | 10a | D | Describe how predictors were handled in the analyses. |  |
|  | 10b | D | Specify type of model, all model-building procedures (including any predictor selection), and method for internal validation. |  |
|  | 10c | V | For validation, describe how the predictions were calculated. |  |
|  | 10d | D;V | Specify all measures used to assess model performance and, if relevant, to compare multiple models. |  |
|  | 10e | V | Describe any model updating (e.g., recalibration) arising from the validation, if done. |  |
| Risk groups | 11 | D;V | Provide details on how risk groups were created, if done. |  |
| Development vs. validation | 12 | V | For validation, identify any differences from the development data in setting, eligibility criteria, outcome, and predictors. |  |
| **Results** | | | | |
| Participants | 13a | D;V | Describe the flow of participants through the study, including the number of participants with and without the outcome and, if applicable, a summary of the follow-up time. A diagram may be helpful. |  |
|  | 13b | D;V | Describe the characteristics of the participants (basic demographics, clinical features, available predictors), including the number of participants with missing data for predictors and outcome. |  |
|  | 13c | V | For validation, show a comparison with the development data of the distribution of important variables (demographics, predictors and outcome). |  |
| Model development | 14a | D | Specify the number of participants and outcome events in each analysis. |  |
|  | 14b | D | If done, report the unadjusted association between each candidate predictor and outcome. |  |
| Model specification | 15a | D | Present the full prediction model to allow predictions for individuals (i.e., all regression coefficients, and model intercept or baseline survival at a given time point). |  |
|  | 15b | D | Explain how to the use the prediction model. |  |
| Model performance | 16 | D;V | Report performance measures (with CIs) for the prediction model. |  |
| Model-updating | 17 | V | If done, report the results from any model updating (i.e., model specification, model performance). |  |
| **Discussion** | | | | |
| Limitations | 18 | D;V | Discuss any limitations of the study (such as nonrepresentative sample, few events per predictor, missing data). |  |
| Interpretation | 19a | V | For validation, discuss the results with reference to performance in the development data, and any other validation data. |  |
|  | 19b | D;V | Give an overall interpretation of the results, considering objectives, limitations, results from similar studies, and other relevant evidence. |  |
| Implications | 20 | D;V | Discuss the potential clinical use of the model and implications for future research. |  |
| **Other information** | | | | |
| Supplementary information | 21 | D;V | Provide information about the availability of supplementary resources, such as study protocol, Web calculator, and data sets. |  |
| Funding | 22 | D;V | Give the source of funding and the role of the funders for the present study. |  |

*Items relevant only to the development of a prediction model are denoted by D, items relating solely to a validation of a prediction model are denoted by V, and items relating to both are denoted D;V. We recommend using the TRIPOD Checklist in conjunction with the TRIPOD Explanation and Elaboration document.
